# Mechanism of Herpes Simplex Virus Inhibition by Antiviral Compounds

**DOI:** 10.1063/4.0000829

**Published:** 2025-10-27

**Authors:** Qing Yao

**Affiliations:** Gilead Sciences, Inc., Foster City, CA, USA

## Abstract

Genital herpes, caused by the Herpes Simplex Virus (HSV), remains a widespread disease with limited treatment options and no preventive vaccines. A key viral protein complex critical to HSV DNA replication has emerged as a promising target for new antiviral drugs. However, the development of more effective treatments has been hindered by a lack of detailed information about the mechanisms of the protein complex inhibition and an incomplete understanding of antiviral inhibitor functions.

This research investigates the inhibition mechanisms of antiviral compounds with proven clinical efficacy against the protein complex. We analyzed the potency of these inhibitors against various viral strains, including those with resistance mutations. Our analysis further clarified the factors influencing their effectiveness. These findings provide valuable insights into the distinct assembly of this viral protein complex and the mechanisms by which these inhibitors function, potentially informing the development of future antiviral therapies for herpesvirus infections.

